# *Raoultella* *terrigena*: Current state of knowledge, after two recently identified clinical cases in Eastern Europe

**DOI:** 10.1002/ccr3.4089

**Published:** 2021-03-31

**Authors:** Nadiia Lekhniuk, Ulbolgan Fesenko, Yaroslav Pidhirnyi, Alicja Sękowska, Olena Korniychuk, Yulian Konechnyi

**Affiliations:** ^1^ Department of Genetics and Biotechnology Ivan Franko National University of Lviv Lviv Ukraine; ^2^ Department of Anesthesiology and Intensive Care Danylo Halytsky Lviv National Medical University Lviv Ukraine; ^3^ Department of Microbiology Ludwik Rydygier Collegium Medicum Nicolaus Copernicus University Bydgoszcz Poland; ^4^ Department of Microbiology Danylo Halytsky Lviv National Medical University Lviv Ukraine

**Keywords:** clinical case, healthcare‐associated infections, *Klebsiella*, literature review, opportunists, phylogenetic analysis, *Raoultellaterrigena*, rare pathogen

## Abstract

*Raoultella terrigena* is a rarely found opportunistic pathogen that can cause healthcare‐associated infections with high mortality. It is important to differentiate it from Klebsiella species.

## Graphical abstract

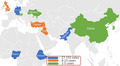

## 1 INTRODUCTION

*Raoultella terrigena (Klebsiella terrigena) is a rarely found opportunistic pathogen. There were reported 363 cases of R. terrigena infection between 1988 and 2021 year. The mortality of this infection about 44%, and in 38.6% of cases, R. terrigena, has MDR antibiotic sensitivity profile. We made a brief literature and clinical case review*.

### 1.1 Our cases presentation

#### 1.1.1 Case No. 1

Patient 1 is a 42‐year‐old female. In September 2018, she was transferred to the intensive care unit (ICU) of the clinical hospital in Lviv due to the development of eclampsia 3 days after a cesarean section and diagnosed with prenatal fetal death of fetus on 39‐40 weeks. The patient's history included cysts of the right kidney, cholelithiasis, and long‐term smoking anamnesis. In the hospital, the patient developed an exacerbation of chronic bronchitis, acute pseudomembranous colitis, and hypoalbuminemia.

At the time of taking the material for microbiological examination (day eight postadmission), the patient was comatose and maintained with artificial lung ventilation (ALV) with urinary and central venous catheters. Treatment consisted of a 6‐day course with imipenem, fluconazole, and nystatin. Five days before sampling, the patient received a blood transfusion. Laboratory results on the day eight indicated: leukocytosis (13.7 × 10^9^/l [N 4‐9] with a left shift—stab neutrophils 20% [N 1‐5], increased ESR [23 mm/h [N 1‐15]), CRP +++ (N `‐`). After disconnecting the patient from the ALV on day nine, the patient presented with a productive cough with mucopurulent sputum discharge.

Microbiological examination of urine on day eight did not reveal the growth of aerobic microbiota. The *R. terrigena* pathogen was isolated from bronchial secretion on day eight at 10^7^ CFU/mL while receiving imipenem therapy. The result of the antibiotic sensitivity test is shown in Table 1 (Case No. 1). Blood culture test for sterility on day sixteen after hospitalization did not detect the growth of aerobic microorganisms. The patient with an improvement in general condition was transferred to the therapeutic department on day 24 after completing a course of antibiotic therapy (ie, imipenem—15 days, vancomycin—10 days, and metronidazole—10 days) and after that with improvement discharged home.

**TABLE 1:** Antibiotic sensitivity test of isolated pathogens

No.	Drug name	Case no. 1	Case no. 2
1	Azithromycin	R	R
2	Amikacin	S	S
3	Amoxiclav	R	R
4	Amoxicillin	R	R
5	Ampicillin	R	R
6	Vancomycin	R	R
7	Gentamicin	S	S
8	Doxycycline	R	iS
9	Imipenem	S	S
10	Colistin	S	S
11	Levofloxacin	R	R
12	Linezolid	R	R
13	Meropenem	iS	iS
14	Maxifloxacin	R	R
15	Netilmicin	S	S
16	Nitroxoline	S	S
17	Norfloxacin	R	R
18	Piperacillin	R	S
19	Sulperasone	iS	S
20	Teicoplanin	R	R
21	Tetracycline	R	R
22	Tigecycline	S	S
23	Tobramycin	S	S
24	Fosfomycin	R	iS
25	Cefazoline	R	R
26	Cefepime	R	R
27	Cefotaxime	R	R
28	Ceftazidime	R	R
29	Ceftriaxone	R	R
30	Cefuroxime	R	R
31	Ciprofloxacin	R	R

Abbreviations: R,resistance; iS, intermediate sensitive; S, sensitive.

[Correction added on 19 April 2021, after first online publication: The sentence ‘Red color, does not metabolize the substrate; green color, metabolizes the substrate; yellow substrate—the results differ in different tests or over time. ’ was published inadvertently and has been removed from the footnote in this current version.]

#### 1.1.2 Case no. 2

Patient 2 is an 18‐year‐old female. The patient was admitted to the ICU of the Clinical Hospital in Lviv in November 2018 with a diagnosis of type 1 diabetes, a stage of decompensation, a degree I coma, and a history of subdural hematoma, periodic episodes of diabetes decompensation due to nonadherence to diet. The patient had a central venous catheter. Additionally, lumbar puncture and tracheal intubation were performed in the patient on day two after hospitalization. The patient received an erythrocyte mass transfusion on day four and a tracheostomy on day five. Body temperature increased from 37.0‐37.8°C at the time of admission to 38.5‐39.0°C on day five after tracheal intubation. At that time, the patient presented with left‐sided hydrothorax (according to Rtg) and pulmonary edema. Ceftriaxone and levofloxacin were administered on day one and fluconazole on day two after hospitalization.

A tracheostomy obtained a bronchial aspirate at day five for microbiological evaluation. At the time of biopsy sampling, the patient had a leukocytosis of 16 × 10^9^/l (N 4‐9) with a left shift—myelocytes 1 (N 0), young (meta‐leukocytes) 2 (N 0‐1), and increased ESR (35 mm/h [N 1‐15]). According to the inoculation results, the *R. terrigena* culture was detected in an amount of 10^6^ CFU/mL. The results of the antibiogram for Patient 2 are shown in Table 1 and compared with those for Patient 1. Targeted pulmonary infection elimination was not possible due to the patient's death from diabetes mellitus decompensation on the second day after receiving the inoculation results. The pathological autopsy revealed a concomitant bilateral small focal lower lobe acinar purulent bronchopneumonia in addition to the underlying disease.

## 2 METHODS

### 2.1 Search strategy and selection criteria

We used PubMed and Google Scholar databases to find all possible information, using search terms “*Raoultella terrigena,” “Raoultella,” and “Klebsiella terrigena,”* from 1988 to 2021 years. Only papers published in English (or with English abstracts) were reviewed. The final reference list was generated based on originality and relevance to the broad scope of this Review.

### 2.2 Pure culture isolation and antibiotic sensitivity test

The isolation and identification of pathogens were carried out in a certified microbiological laboratory of the Department of Microbiology at the Danylo Halytsky Lviv National Medical University, Ukraine. A Bacteriological Culture Method was used to isolate a pure pathogen culture. Antibiotic Sensitivity Test was made using M100 Performance Standards for Antimicrobial Susceptibility Testing.^1^ Microbial cultures were disposed of according to standard laboratory biosafety protocols.

### 2.3 Biochemical identification

The pathogen identification was performed using the MIKRO‐LA‐TEST ENTERO kit (ErbaLachema) in five technical repetitions and using the corresponding codebook.^2^

### 2.4 Phylogenetic analysis

**FIGURE 1:**
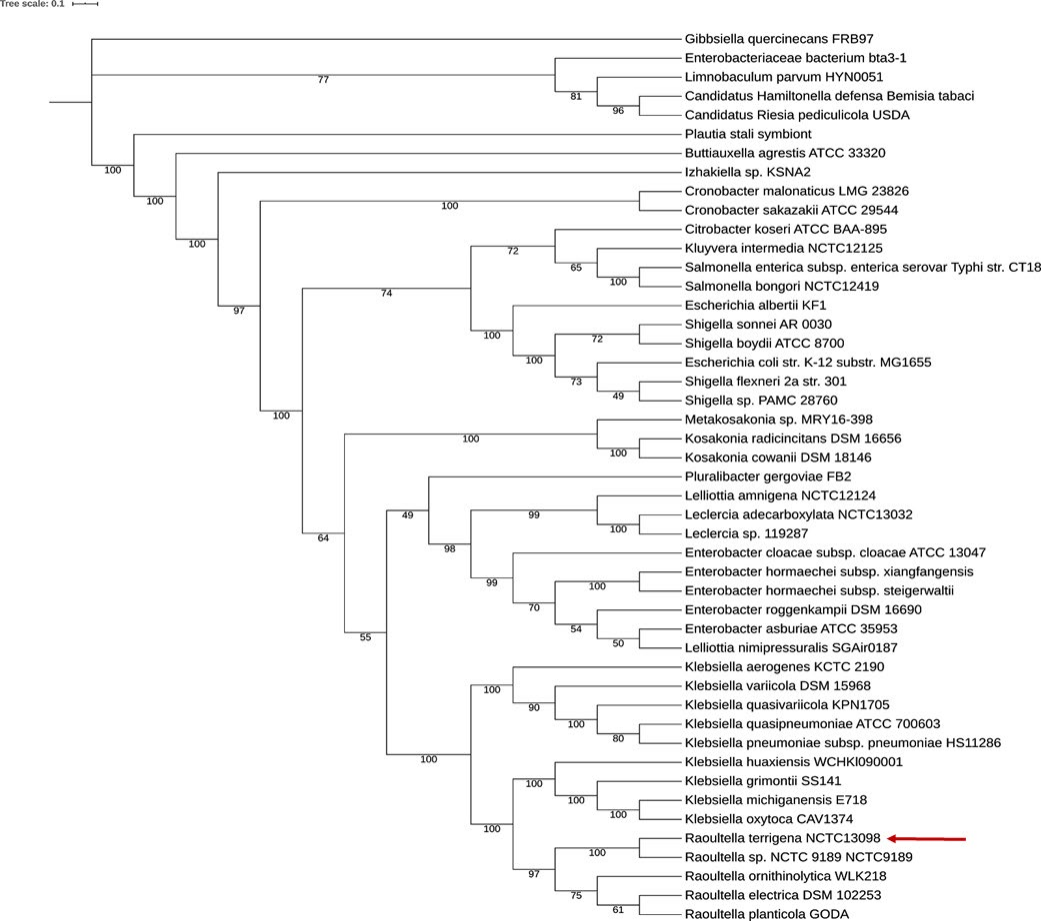
Cladogram of the *Enterobacteriaceae* family. Figures—percentages of the topology reliability. Ultrafastbootstrap (n = 1000) was used as a validation method [Correction added on 19 April 2021, after first online publication: Figures 1 and 3 were inadvertently swapped and they have now been placed with the right captions in this current version.]

Was aligned the sequenced genomes of members of the *Enterobacteriaceae* family to compile a phylogenetic tree of the Enterobacteriaceae family (Figure 1). The genomes were loaded manually from the GenBank database. Species of the *Klebsiella* and *Raoultella* genera were all loaded, considering the completeness of the genomes. For the phylogeny markers identification, the Phylophlan2 program, the improved version of the original Phylophlan software,^3^ with the amphora2 database (136 proteins) was used. Default parameters of the Phylophlan2, which we applied, included a diamond algorithm for homology search of the 136 proteins in selected genomes and MAFFT algorithm with auto parameters for aligning newly formed concatemers.^4^, ^5^ For the further construction of phylogeny, the CIPRES supercluster was used, namely, IQTree software with standard settings and the ultrafast bootstrap approximation as a topology reliability criterion (n = 1000).^6^, ^7^

From the cladogram (Figure 1), we can see that the *Raoultella* genus bacteria form a distinct group from *Klebsiella*. Besides, the *Klebsiella* and *Raoultella* sequences represent the most distantly related groups when compared with the other bacterial sequences.

## 3 DISCUSSION

### 3.1 Discussion of case 1 and case 2

*Raoultella terrigena* infection can damage different organs (lungs, wounds, and general septic infection), especially in patients with chronic diseases. It was interesting to know in case No. 1 that administration of imipenem during 5 days before biological sampling for inoculation did not affect the inoculation of the pathogen, even though the cultured *R. terrigena* strain was sensitive to imipenem, suggests a constant source of infection for the patient, which could potentially be due to the device tubes, as previously described by Hurrel et al showing the growth of the pathogen *R. terrigena* was associated with tubes for newborn feeding.

**FIGURE 2:**
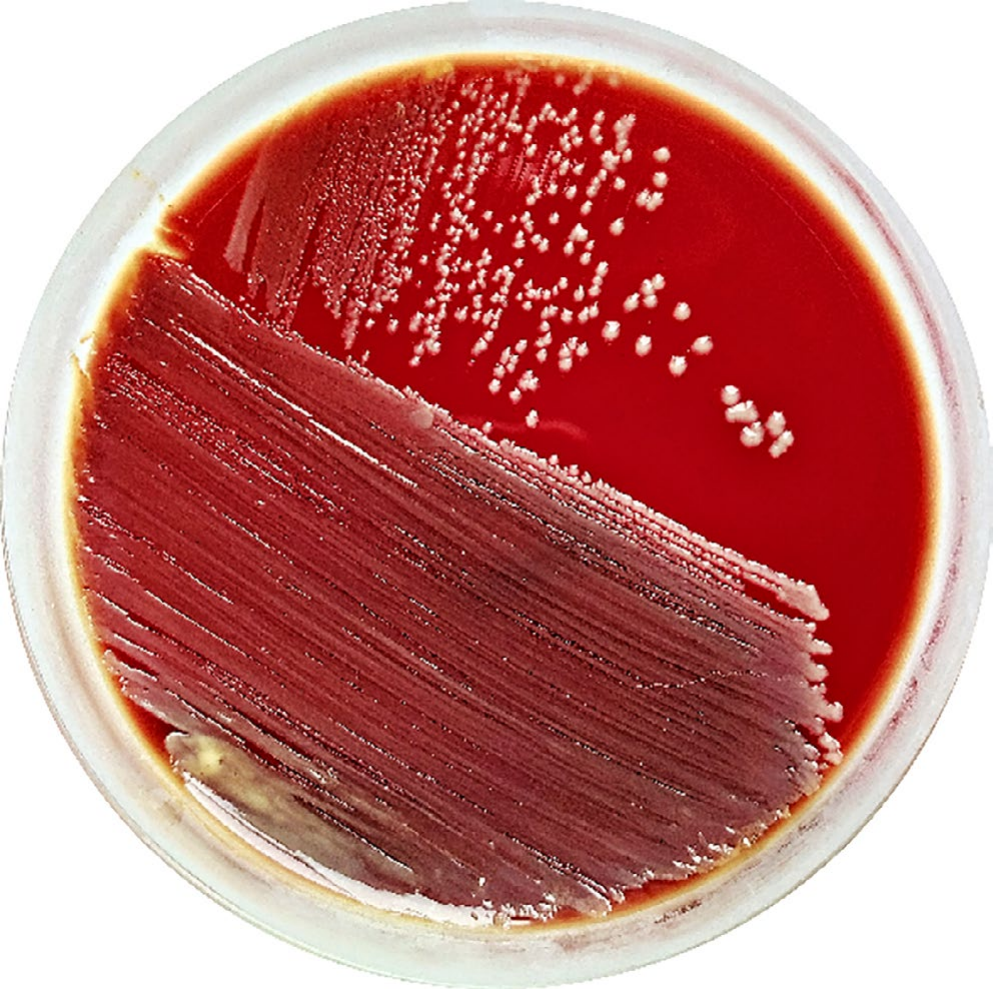
*Raoultella terrigena* (case 1) stock culture on blood agar, using streak plate technique for single colony isolation [Correction added on 19 April 2021, after first online publication: Figures 2 and 4 were inadvertently swapped and they have now been placed with the right captions in this current version.]

**FIGURE 3:**
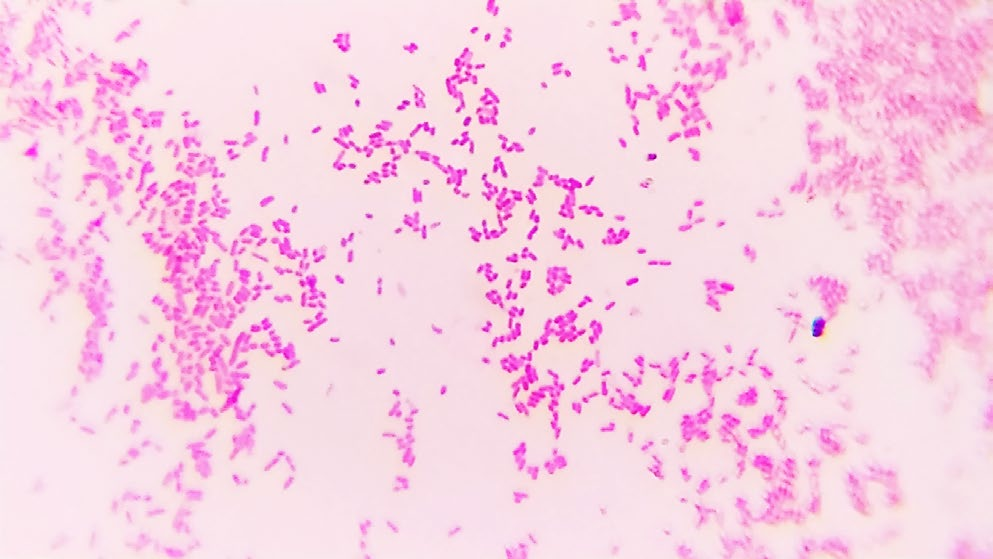
Light microscopy of *R. terrigena* case 1 (Gram staining, 10 × 100 magnification). Gram‐negative rod‐shaped bacteria, 1‐2 μm, typical for *Enterobacteriaceae* family

In both cases of this study, *R. terrigena* isolates were unpretentious to the cultivation conditions; it is cultured on ordinary nutrient media (beef‐extract agar, blood agar, but worth growing in Endo (lactose‐containing) medium) at 37°C, hemolysis (‐), without a specific smell or pigment. Colonies are not specific. Figures 2 and 3 show *R. terrigena* colony on blood agar. Biochemical identification results are shown in Table 2.

**TABLE 2:** *Raoultella terrigena* (case 1 and case 2) biochemical properties profile

Case no. 1																		
OXI	URE	LAC	GAL	ARG	MAN	MLT	ORN	TRE	CEL	LYS	XYL	SUC	AAM	ARA	INO	BGL	AGA	GGT
																		
NAG	BGA	PHS	SCI	MAL	ESL	IND	PHE	VPT	H2S	ONP	SOR	ADO	RHA	MLB	RAF	DUL	MAL	GLU
																		

Case no. 2																		
OXI	URE	LAC	GAL	ARG	MAN	MLT	ORN	TRE	CEL	LYS	XYL	SUC	AAM	ARA	INO	BGL	AGA	GGT
																		
NAG	BGA	PHS	SCI	MAL	ESL	IND	PHE	VPT	H2S	ONP	SOR	ADO	RHA	MLB	RAF	DUL	MAL	GLU
																		

Note

Abbreviations: AAM, acetamide utilization; ADO, acid from adonitol; AGA – α‐glucosidase; ARA, acid from L‐arabinose; ARG, arginindihydrolase; BGA, β‐galactosidase; BGL, β‐glucosidase; CEL, acid from cellobiose; DUL, acid from dulcitol; ESL, ecsulin hydrolysis; GAL, acid from galactose; GGT, γ‐glutamyle‐transferase; GLU, acid from glucose; H2S, hydrogen sulfide production; IND, indole production; INO, acid from myo‐inositol; LAC, acid from lactose; LYS, lysindecarboxylase; MAL, malonate utilization; MAL, malonate utilization; MAN, acid from mannitol; MLB, acid from mellibiose; MLT, acid from maltose; NAG, N‐acetyl‐glucosaminidase; ONP, β‐galactosidase (ONPG); ORN, ornithindecarboxylase; OXI, oxidase; PHE, phenylalanindeaminase; PHS, phosphatase production; RAF, acid from raffinose; RHA, acid from rhamnose; SCI, citrate utilization; SOR, acid from sorbitol; SUC, acid from saccharose; TRE, acid from trehalose; URE, urea hydrolysis; VPT, acetonin production; XYL, acid from xylose.

Red color, does not metabolize the substrate; green color, metabolizes the substrate; yellow substrate—the results differ in different tests or over time.

The delayed onset of infection in the patients (ie, at least 48 hours after hospital admission), and the fact that both patients were in the same ICU (at different times) and on the ALV with similar antibiotic response profiles (Table 1) suggest the involvement of nosocomial transmission of the infection for the two clinical cases shown here. We speculate that *R. terrigena* may play an essential role in device‐associated nosocomial opportunistic infections.^8^

A regular study of the microbial landscape among patients in this ICU was not carried out, and the search for a possible source of infection in the department or hospital is not routinely done in these cases and is usually at the discretion of the medical facility where the issue occurs. These two clinical cases discussed here highlight the need to establish in Ukraine a more standardized system to record and investigate nosocomial infections with potentially undesirable public health effects.

### 3.2 *Raoultella terrigena* short overview

The *Raoultella* genus was separated from *Klebsiella* in 2001 basing on the analysis of 16S rRNA sequences and the *rpoβ* gene.^9^ However, difficulties with the correct identification of the pathogen still arise, which may lead to underestimating its presence and inaccuracies in determining its pathological role. A retrospective study of 240 blood samples from the collection of the Ramón y Cajal University Hospital laboratory in Madrid (Spain, 2015), with bacteria of the *Klebsiella* genus observed, has shown that 11 of them actually contained bacteria of the *Raoultella* genus. *Raoultella planticola* and *R. ornithinolytica* cause most *Raoultella* bacteremia cases. *Raoultella terrigena* and *R. electrica* species also belong to this genus. Typical habitats for these pathogens are freshwater, plants, and soil. *Raoultella* acts as a conditionally pathogenic component of the microbiota, in most cases causing bile duct infections. Since the genome of representatives of this genus contains the class A *β*‐lactamase gene, a natural universal resistance to ampicillin is observing for all *Raoultella* isolates.^10^ Now 80 cases of bacteremia secondary to *Raoultella spp*, with various primary infection sources, are known.^11^

In general, these bacteria are sensitive to amoxicillin, piperacillin, piperacillin‐tazobactam, cephalosporins of 2‐4 generations, carbapenem, aminoglycosides, trimethoprim/sulfate methoxazole, and tigecycline. Like *Klebsiella, Raoultella* can acquire antibiotic resistance genes, leading to an increase of the multidrug‐resistant (MDR) isolates. Recent work describing infections caused by *Raoultella* has shown that these bacteria are characterized by various resistance mechanisms, especially those involving sulfonamides and quinolone and antibiotics of the *β*‐lactam spectrum.^12^, ^13^ Resistance genes for such drugs are localized in bacterial plasmids; they are not a constitutive component of their genome.

The sensitivity of *Raoultella* to disinfectants, as well as to antibiotics, was also studied and revealed ambiguous results. In particular, studies on various isolates of *R. planticola* showed growth retardation under the influence of sodium hypochlorite, benzalkonium chloride, n‐hexadecyltrimethylammonium chloride, and 1‐hexadecylpyridinum chloride. In contrast, the other isolates were resistant to most of the antibiotics, disinfectants, and the effects of metals such as aluminium and heavy metals such as iron, nickel, and others.^14^, ^15^

Bacteria of the *Raoultella* genus do not show a high level of virulence. The main virulence factors of these bacteria are lipopolysaccharides (O‐antigen), polysaccharide capsules (K‐antigen), adhesins (fimbriae class 1 and class 3), siderophores (enterobactin and aerobactin), tetrodotoxin, different hydrolytic enzymes, bacteriocins, such as raoultellin L *Raoultella* isolates are also able to form biofilms. It has been shown that processing crops, particularly tomatoes, with a suspension of *R. terrigena* have antiparasitic activity and can be used in agricultural biotechnology.^16^ *Raoultella terrigena* could be isolated from the intestine of pufferfish,^17^ milk^18^ and may be used in biotechnology as 2,3‐Butanediol produser.^19^

*Raoultella terrigena* are gram‐negative nonmotile capsule forming bacteria belonging to the *Enterobacteriaceae* family. They are facultative anaerobes having both respiratory and enzymatic types of metabolism. Next to *R. electrica*, infections caused by *R. terrigena* are rare, and the identification and study of cases of infection with this bacterium are a promising and important area of modern clinical microbiology because the role of *R. terrigena* in the pathogenesis of disease is underestimated.^20^

In 2019, the genome sequencing of two clinical strains of *R. terrigena* (NCTC 13097 and NCTC 13098) isolated from the mucous membrane of a child's tonsils and a patient with chronic bronchitis was completed. This pathogen's genome size is 5.5‐6 Mb, and its GC content is about 57%. One of the strains also contained an 86 kb plasmid. Bioinformatic analysis of the *R. terrigena* genome identified 5500 protein‐coding sequences in addition to 85 tRNA and 25 rRNA sequences.^21^

### 3.3 *Raoultella terrigena (Klebsiella terrigena)* infection previous cases review

**FIGURE 4:**
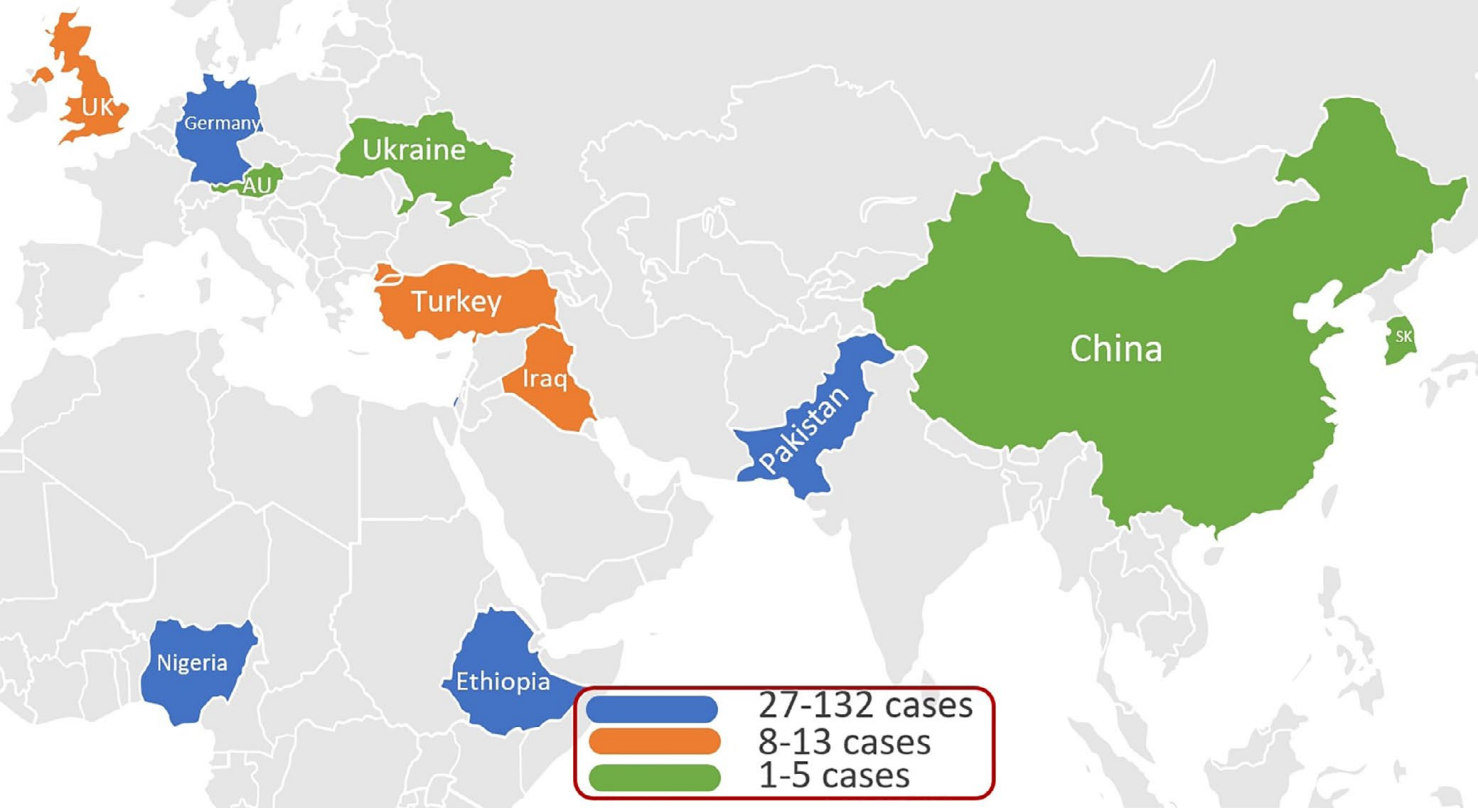
The prevalence of *R. terrigena (Klebsiella terrigena*) infection reported cases in the world from 1988 to 2021

There are 363 reported cases of *Raoultella (Klebsiella) terrigena* in humans from 1988 to 2021, including 2 report cases in this study. 132 cases (36,3%) of *K. terrigena* in Germany, 69 cases (19,0%) from Pakistan, 56 cases (15.4%) from Palestine, 36 cases (9,9%) from Ethiopia, 27 cases (7.4%) from Nigeria, and 43 cases (11,8%) from another countries (United Kingdom, Turkey, Iraq, South Korea, Ukraine, Austria, and China (Figure 4 and Table 3). In 140 reported cases (38.6%), *R. terrigena* were MDR. In 13.6% of cases, *R. terrigena* was isolated from human feces without any clinical symptoms.

**TABLE 3:** Comparative characteristics of *Raoultella terrigena (Klebsiella terrigena*) infection clinical cases in the world from 1988 to 2021

Author (ref)	Country	Year	No of cases	Medical history background/testing biomaterial	Source of infection	Treatment or sensitivity to antibiotics	Outcome
^25^	Germany	1988‐1990	10	*Klebsiella terrigena* Respiratory tract (8), urine (1), wound infection (1)	Unknown	Non‐MDR	No information
^24^	Germany	1990	50	*Klebsiella terrigena* Fecus	Unknown	Non‐MDR	No information
^35^	South Korea	1999	5	Respiratory tract (3), blood (1), aspiration (1),	Unknown	Non‐MDR	No information
^26^	Germany	2000	72	50 cases (feces) of *Klebsiella terrigena* from healthy people + 22 cases from clinical cases (15 respiratory secretions, 1 blood culture, 2 urine, 4 wounds)	Unknown	Non‐MDR	No information
^36^	Palestine (Gaza)	2005	56	Blood from newborn in ICU	Unknown	MDR	No information
^27^	Austria	2007	1	45 y old male. Liver transplantation due to hepatitis C.	Unknown	Piperacillin/tazobactam	Death
^28^	United Kingdom	2009	13	Feeding tubes were collected from two neonatal intensive care units	Unknown	Non‐MDR	No information
^29^	United Kingdom	2011	1	69 y old male. Whipple's procedure due to pancreatic cancer	Unknown	Imipenem, piperacillin/tazobactam	Successful treatment
^37^	Iraq	2011	7	Wound and blood samples	Unknown	No information	No information
^38^	Iraq	2014	1	Urine (1) in child	Unknown	MDR	No information
^39^	Nigeria	2014	27	Swab (4), urine (15), blood (2), CSF (1), sputum (3), pus (2)	Unknown	No information	No information
^22^	Pakistan	2013‐2018	58	Respiratory tract (13), urinary tract (12), blood (12)	Unknown	Meropenem plus colistimethate/ >80% MDR	44.7% of mortality
^30^	Turkey	2015	13	Premature newborn. Mother had a cesarean section due to threatened preterm labor.	Nosocomial	Cephaperazone/sulbactam	Successful treatment
^34^	China	2016	1	63 y old male. Painful abscess on the right thumb after injury.	River water	Imipenem	Successful treatment
^31^	Ethiopia	2016	36	Urine	Unknown	MDR	No information
^32^	Pakistan	2016‐2019	8	Blood	Unknown	No information	No information
This study, case 1	Ukraine	2018	1	42 y old female. Had cesarean section due to eclampsia, diagnosed with antenatal death of fetus. Pleurisy, cerebral edema, multiple organ dysfunction syndrome.	Nosocomial	Imipenem	Successful treatment
This study, case 2	Ukraine	2018	1	18 y old female. Type 1 diabetes, intracerebral hemorrhage, retinopathy, multiple organ dysfunction syndrome, and chronic kidney disease. Case of subdural hematoma.	Nosocomial	Ceftriaxone, levofloxacin	Death
^20^	Pakistan	2019	1	30 y old female. Uncontrolled diabetes and a history of recurrent miscarriages presented with altered mentation and sepsis.	Unknown	Fosfomycin, tigecycline, cotrimoxazole	Death
^20^	Pakistan	2019	1	63 y old female. Diabetes, hypertension, rheumatoid arthritis, and iatrogenic Cushing's syndrome due to steroid self‐medication	Unknown	Fosfomycin, tigecycline, cotrimoxazole, fluconazole	Death
^20^	Pakistan	2019	1	63 y old female. Hypertension, ischemic heart disease, chronic obstructive pulmonary disease, and chronic kidney disease. History of lower respiratory tract and urinary tract infections	Unknown	Fosfomycin	Successful treatment

*Raoultella terrigena* infection showed high mortality (44.4%), 4 patients from 9 separately reported clinical cases with presented outcome information have died (Table 3). Similar mortality levels (44.7%) have been reported in Pakistan during 2013‐2018 years.^22^ In our case, 1 of our study patient had pregnancy, eclampsia, and long‐term smoking anamnesis; in case 2, the patient had decompensation of type 1 diabetes, so we may say both patients were immunocompromised.^23^

Retrospective analysis of isolation of 50 strains of *K. terrigena* from human feces was provided in 1990 at the University of Kiel (Germany). Still, it was no reported about its clinical significance, so the hypothesis of *K. terrigena* as a part of normal human microbiota was assumed.^24^ And already in 2 years, the same authors reported about 10 clinical cases of *K. terrigena* infections.^25^ 50 cases (feces) of *Klebsiella terrigena* were isolated from healthy people and 22 cases from clinical cases (15 respiratory secretions, 1 blood culture, 2 urine, and 4 wounds) in 2000 in UK.^26^

At present, there are few separately, and detail described cases of *R. terrigena* infection in the world, which prevent us from establishing a specific clinical symptomatic profile for this pathogen or a clear outline of the disease course, leaving many open questions and information gaps. The variety of possible clinical manifestations does not differ from those that are characteristic of representatives of the *Enterobacteriaceae* family or *Klebsiella* genus.

From 1990 to 2021, there have been no reported and described clinical cases of *Raoultella terrigena* infection in Ukraine. However, an overview of worldwide *R. terrigena* prevalence, shown in Figure 1, indicates that some cases of *R. terrigena* were seen in Europe for that period. Furthermore, Table 3 details *R. terrigena* infection cases as of 2021, summarizing the patients' clinical history, including the source of infection and treatment regimen. There was no correlation between geography, environmental conditions, seasons of the year, patients, or tissue target in described cases in Table 3.

The first case of confirmed infection with *R. terrigena* (as *Raoultella*, not *Klebsiella*) dates back to 2007 (Austria), where the bacterium was identified from a fatal case of endocarditis in a 45‐year‐old liver transplant recipient with hepatitis C. Despite the 10‐day piperacillin/tazobactam therapy, the patient developed pneumonia, progressing into the infectious endocarditis.^27^

An inter‐center study was conducted in the UK in 2009 to identify a spectrum of pathogens that can colonize in tubes for feeding infants. The results showed that more than half of the tubes in the neonatal resuscitation and intensive care units grew colonies of the *Enterobacteriaceae* family, including 10% of the isolates being *R. terrigena*. However, there were no infection cases with this pathogen, or they were not correctly identified.^28^

The next confirmed *R. terrigena* incident was notified in the UK. This case led to the development of sepsis coursed by *R. terrigena* in a 69‐year‐old man after resection of the pancreas (Whipple procedure). The patient was cured after piperacillin‐tazobactam treatment.^29^

A case of *R. terrigena* infection of a premature baby was reported in Turkey for the first time in 2015. The baby was placed in the intensive care unit (ICU) and released after successful 10‐day treatment with cefoperazone/sulbactam.^30^

Thirty six cases of urinary tract infections in the ICU in Ethiopia in 2016 retrospectively were described in 2018. All 36 strains of *R. terrigena* were MDR.^31^ And 8 cases were reported in Pakistan as bacteriemia.^32^ 58 cases of *R. terrigena* in another hospital in Pakistan were reported during 2013‐2018 years, 12 patients of which were colonized without clinical symptoms.^22^ There were isolated 2 strains of *R. terrigena* from the gallbladders in patients with gallstones after cholecystectomy,^33^ but without connection between stone formation or inflammation and isolated pathogens.

An infection of a 63‐year‐old Chinese farmer was reported in 2016 after being admitted to the hospital complaining of painful spasms in his wounded finger. Sequencing of the bacterial 16S rRNA and the pathogen's constitutive genes identified a focus of *R. terrigena* infection. A likely source of infection was river water used to wash the injured hand. The infection was eliminated after 10 days following treatment with imipenem twice daily.^34^

The latest evidence of infection with *R. terrigena* at the time of writing this article came from three clinical cases in the Indus Health Network, Pakistan. The authors describe the following case histories of patients admitted to the clinic in 2018‐2019. The first case concerns a 30‐year‐old woman with uncontrolled diabetes and a history of skilled miscarriage, accompanied by impaired consciousness and sepsis. This patient was hospitalized with general weakness, anorexia, and periodic fevers. Besides, *R. terrigena* was found in the patient's blood and urine and was treated with fosfomycin‐tigecycline cotrimoxazole therapy. However, the patient developed hypocalcemia complications resulting in cardiac arrhythmia, leading to death on the 14th day of hospitalization. The second case, a 63‐year‐old woman with diabetes, hypertension, rheumatoid arthritis, and Cushing's syndrome, was admitted to the hospital with generalized body swelling. Among other bacteria, *R. terrigena* sensitive only to colistin and cotrimoxazole was found in the woman's peripheral blood. Despite the successful antibiotic therapy for all infectious agents, the woman died of disseminated intravascular coagulation and multiple organ failure on the 26th day of hospital stay. The third patient was a 63‐year‐old woman with hypertension, ischemia, chronic obstructive pulmonary disease, and secondary renal failure, who underwent a 2‐month treatment for infections of the lower respiratory tract and the genitourinary system at the time of transfer to the above‐mentioned medical network. Three pathogens have been detected in this patient: *Staphylococcus epidermidis*, *Pseudomonas spp*. and *R. terrigena*. Therapy was performed with vancomycin, ceftazidime, and fosfomycin, respectively. The woman was cured of all three infections and transferred to a regional hospital due to other complicated medical conditions.^20^

## 4 CONCLUSION

We analyzed 363 cases of *R. terrigena* infection, including a description of 2 our cases.

*Raoultella terrigena* is an opportunistic pathogen with a high level of mortality (till 44%), which could lead to infection process with endogenic source of infection (feces and bile) as well as exogenic souse (water, milk, and soil) and also may be associate with healthcare‐associated infection. The real level of *R. terrigena* infection could be higher due to distinguish complication of *R. terrigena* from *Klebsiella,* that is why most studies were retrospective.

It is not clear could the *R. terrigena* be a part of the normal microbiota of the human intestine or its asymptomatic carriers of the pathogen.

## 5 STUDY LIMITATIONS

One of the best pathogen identification methods is 16S rRNA gene sequences, but we did not have the technical opportunity for it. Identification by the pathogen's biochemical properties is also possible using specialized test systems and kits if there is no opportunity for 16S rRNA gene sequences.^34^

## 6 FURTHER RESEARCH PROSPECTS

We plan to investigate the sensitivity of *R. terrigena* to in‐hospital disinfectants, in particular to those used for sterilization of permanent components of lungs ventilators. And we plan to make 16S rRNA gene sequences of these strains. And it is interesting to analyze the correlation between age, gender, and living condition and *R. terrigena* infection and Raoultella as part of the human body's normal microbiota.

## CONFLICT OF INTEREST

All authors declare no conflict of interests.

## AUTHOR CONTRIBUTIONS

NL: involved in original draft preparation. UF: involved in clinical part. YP: involved in supervision. AS: involved in review and editing. OK: involved in project administration. YK: involved in idea, visualization, and investigation.

## ETHICAL APPROVAL

Protocol number 6 of Ethics Committee of research, experimental development, and scientific works of Danylo Halytsky Lviv National Medical University (Ukraine) behalf of 25 June 2018.

## Data Availability

The information used and/or analyzed during this case report is available from the corresponding author on reasonable request. If requested (please contact yulian.konechnyi@gmail.com ).
